# Variability of the Penn upper motor neuron score in amyotrophic lateral sclerosis: need for a revised score

**DOI:** 10.1007/s00415-025-12895-7

**Published:** 2025-02-15

**Authors:** Anna B. Jacobsen, Gaia Fanella, Mamede de Carvalho, Martin Koltzenburg, Miguel Oliveira Santos, Bülent Cengiz, Jakob Blicher, Izabella Obál, Mia B. Heintzelmann, Wilfred Nix, Jean-Philippe Camdessanché, Anders Fuglsang-Frederiksen, Hatice Tankisi

**Affiliations:** 1 Department of Clinical Neurophysiology, Aarhus University Hospital, Palle Juul-Jensens Boulevard 165, J209, 8200 Aarhus, Denmark; 2 Department of Clinical Medicine, Aarhus University, Aarhus, Denmark; 3 Institute of Physiology, Centro de Estudos Egas Moniz, Faculdade de Medicina, Instituto de Medicina Molecular João Lobo Antunes, Universidade de Lisboa, Lisbon, Portugal; 4 Department of Neurosciences and Mental Health, ULS Hospital de Santa Maria, Lisbon, Portugal; 5 Department of Clinical Neurophysiology, National Hospital for Neurology and Neurosurgery, Queen Square, London, UK; 6 Department of Neurology, Faculty of Medicine, Gazi University, Beşevler, Ankara, Turkey; 7 Department of Neurology, Aalborg University Hospital, Aalborg, Denmark; 8 Department of Clinical Medicine - Center of Functionally Integrative Neuroscience, Aarhus, Denmark; 9 Department of Neurology, Aarhus University Hospital, Aarhus, Denmark; 10 Department of Neurology, Johannes Gutenberg University, Mainz, Germany; 11 Department of Neurology, University Hospital of Saint-Etienne, Saint-Etienne, France

**Keywords:** Motor neuron disease, Diagnosis, Upper motor neuron, Reliability

## Abstract

There is a need for a consensus on a clinical scale for evaluating upper motor neuron (UMN) burden in amyotrophic lateral sclerosis (ALS) to improve consistency in clinical diagnosis, research and monitoring of disease progression. The Penn upper motor neuron score (PUMNS) is the most commonly published scale, however, the reliability of the scale has only been evaluated in a single study involving two raters. The objective of this study was to evaluate the inter-rater reliability of the PUMNS in ALS patients among multiple raters, and to discuss an updated UMN score including the signs with the highest inter-rater reliability. This study included seven ALS patients (mean age: 71 ± 11.5, six males, one female). Each patient was evaluated with the PUMNS by eight raters from different centers blinded to previous observations. The intra-class correlation coefficient (ICC) was calculated to assess the inter-rater reliability of the total PUMNS. The inter-rater reliability of the binary subscores was assessed with Gwet’s AC1 coefficient. The inter-rater agreement for the total PUMNS yielded an ICC of 0.81 (95% CI 0.56;0.96). Items with the highest inter-rater reliability included Hoffman's sign, Babinski's sign, clonus and deep tendon reflexes, while the facial reflex (Gwet’s AC1 −0.038 (95% CI −0.25,0.18)) and crossed adduction (0.18 (95% CI (−0.32,0.67)) had the lowest inter-rater reliability. In conclusion, PUMNS demonstrated good inter-rater reliability overall, while some of the subscores had poor inter-rater reliability. Based on this, we call for an updated UMN score to enhance diagnostic accuracy and research consistency in ALS.

**Supplementary Information:**

The online version contains supplementary material available at 10.1007/s00415-025-12895-7.

## Introduction

In 2020, the Gold Coast criteria were introduced, simplifying the diagnostic criteria for amyotrophic lateral sclerosis (ALS) [1]. According to these criteria, a diagnosis can be made if there are findings of upper motor neuron (UMN) and lower motor neuron (LMN) lesions in one region, or LMN lesions in two regions, along with evidence of disease progression, and after excluding other disorders that mimic ALS. While clinical LMN symptoms and signs can be reliably supported by results of nerve conduction studies and electromyography (EMG), the identification of UMN lesions continues to be largely based on clinical observations which may involve subjective judgment, that can introduce inconsistency. It is well described that it can be challenging to clinically identify UMN signs in ALS patients [2–4]. The reasons for this are not fully understood but may involve complex pathophysiological abnormalities in descending and segmental spinal circuitry and loss of LMN, which are the final common pathway of the abnormal reflexes [2, 5]. Currently, the most widespread paraclinical methods for quantifying UMN abnormalities are magnetic resonance imaging studies [6] and transcranial magnetic stimulation (TMS) [7]. However, there is limited knowledge of how consistent clinical findings of UMN involvement in ALS are reflected by paraclinical abnormalities in imaging and physiological endpoints. Yet, this is essential for a better understanding of the pathophysiology of ALS.

Various UMN scores (UMNS) exist, but there is currently no consensus on which type of scale performs best for the clinical quantification of UMN signs in ALS [8]. Consequently, different approaches and versions of scales for UMN assessment are used [9–15]. This lack of a standardized approach makes it challenging for clinicians and researchers to consistently assess and compare UMN symptoms. A validated and reliable UMNS would help solve the challenge with potential inconsistencies in patient care and research outcomes.

The Penn Upper Motor Neuron Score (PUMNS) has evolved as one of the most widely used tools. It was developed at the Penn Comprehensive ALS Center at the University of Pennsylvania with data from approximately 1800 patients with ALS and primary lateral sclerosis (PLS) [16]. The scale draws from previously established UMNS in the literature [10, 13] and includes elements from two validated scales: the Ashworth Spasticity Scale [17] and the CNS Lability Scale (CNS-LS) for assessing pseudobulbar affect [18]. PUMNS has a range from 0–32 points and incorporates measures of reflexes, pseudobulbar affect and muscle tone. The inter-rater reliability of the PUMNS has only been evaluated in a single study from 2020 [19]. In this study, 30 patients with ALS were evaluated by two raters. There was a good correlation between PUMNS of the two different raters. Using Cronbach alpha, the inter-rater reliability was reasonable with subscore correlation coefficients between 0.68 and 0.85. However, a limitation of the study was that the evaluations were done by only two raters from a single center.

We hypothesize that when the PUMNS is applied by raters from different centers, simulating a clinical setting more accurately, the inter-rater variability will increase. Thus, the objective of this study was to evaluate the inter-rater reliability of the PUMNS in ALS patients among multiple raters from multiple international centers. A secondary aim was to identify the elements with the highest inter-rater reliability to initiate a discussion on updating the UMNS.

## Methods

### Participants

Seven patients with ALS were recruited from the Neurology departments in Aarhus and Aalborg. These patients met the diagnosis of ALS as defined by the Gold Coast criteria [1]. Exclusion criteria included previous central or peripheral nervous system diseases other than ALS, advanced disease stage/bed-bound status, symptoms of respiratory distress and dementia. Three of the patients were on analgesics, such as paracetamol, tramadol, and ibuprofen. None of the patients were receiving treatment for spasticity. Five of the patients received treatment with riluzole. All observations described below were conducted on the same day and lasted approximately 1.5 to 2 h per patient including breaks for patient resting. The study was performed in relation to a European Standardised Tool to Evaluate Electrodiagnostic Methods (ESTEEM) meeting.

### Measures

The patients were evaluated by the same rater using the revised ALS Functional Rating Scale (ALSFRS-R) [20], the Center for Neurologic Lability Scale (CNS-LS) [18], and muscle strength assessed with the Medical Research Council (MRC) score for the following movements: neck flexion and extension, shoulder abduction and adduction, elbow flexion and extension, wrist flexion and extension, finger flexion and extension, thumb abduction, index finger abduction, 5th finger abduction, hip flexion, knee flexion and extension, ankle dorsiflexion and plantarflexion, and hallux flexion and extension, resulting in a maximum total score of 180. Each patient was evaluated with the PUMNS by eight different raters. All items were assessed by all raters, except for the CNS-LS, which was tested by only one rater in the native language to avoid language barriers. Thus, the maximum score was 31. All raters were specialists in Neurology from six different centers across five countries, including Denmark, Turkey, France, the United Kingdom, and Portugal. The raters had a mean (± SD) of 17.8 ± 9.0 years of experience with ALS and had completed their training in Neurology 16.4 ± 10.7 years ago. The raters were blinded to each other's scores. They received a copy of the published PUMNS (see supplemental material) on the day of the study. All raters were familiar with the PUMNS but received no further instructions on how to execute and score the examination of the patients. After the examination, the examiners completed a questionnaire regarding their experience with and interpretation of each item on the PUMNS.

### Statistical analysis

All analyses were conducted using STATA 17.0. Parametric data are represented as the mean ± standard deviation and non-parametric data as the median and interquartile range. Intra-class correlation coefficient (ICC) for two-way random-effects model with absolute agreement was calculated to assess the inter-rater reliability of the total PUMNS. Gwet’s AC1 coefficient [21, 22] was used to evaluate inter-rater reliability of the binary subscores. Results with p-values < 0.05 were considered significant.

## Results

### Patient demographics

Among the seven patients included in the study, the mean age (± SD) was 71 ± 11.5 years and the mean disease duration was 38.3 ± 47.6 months. The cohort consisted of six males and one female, including three patients with bulbar onset and four with spinal onset. The mean ALSFRS-R was 36.1 ± 5.7, the mean MRC score was 158.6 ± 9.4 and the mean CNS-LS was 17.6 ± 8.8. Individual scores for each patient are summarized in Table 1.

**Table 1:** Mean Penn Upper Motor Neuron Score (PUMNS), standard deviation (SD) and range of PUMNS, the revised ALS Functional Rating Scale (ALSFRS-R), Medical Research Council (MRC) score and the Center for Neurologic Lability Scale (CNS-LS) for each patient

Patient	PUMNS (mean)	SD	Range (min;max)	ALSFRS-R	MRC score	CNS-LS
1	13.63	2.72	11;18	33	171	33
2	8.13	3.80	4;14	43	162	15
3	8.50	2.98	4;12	41	164	14
4	6.13	2.36	3;10	41	160	7
5	4.88	3.52	0; 9	34	161	10
6	19.38	3.38	16;26	34	148	23
7	21.63	3.25	16;27	27	144	21

### Inter-rater reliability

**Fig. 1:**
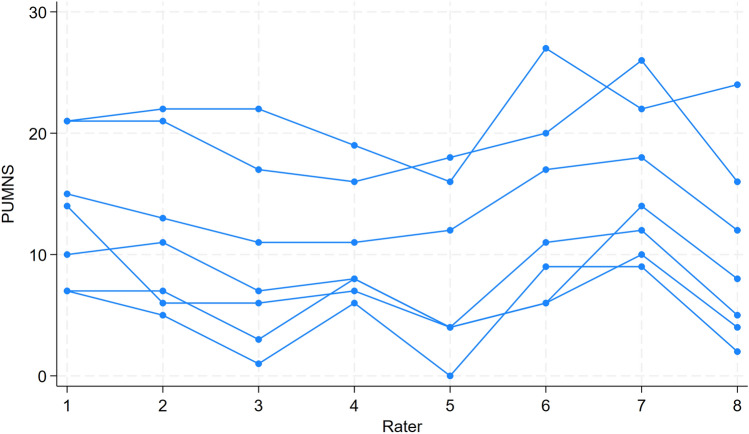
The total Penn Upper Motor Neuron Score (PUMNS) given by each of the eight raters, with each line representing a different patient

**Fig. 2:**
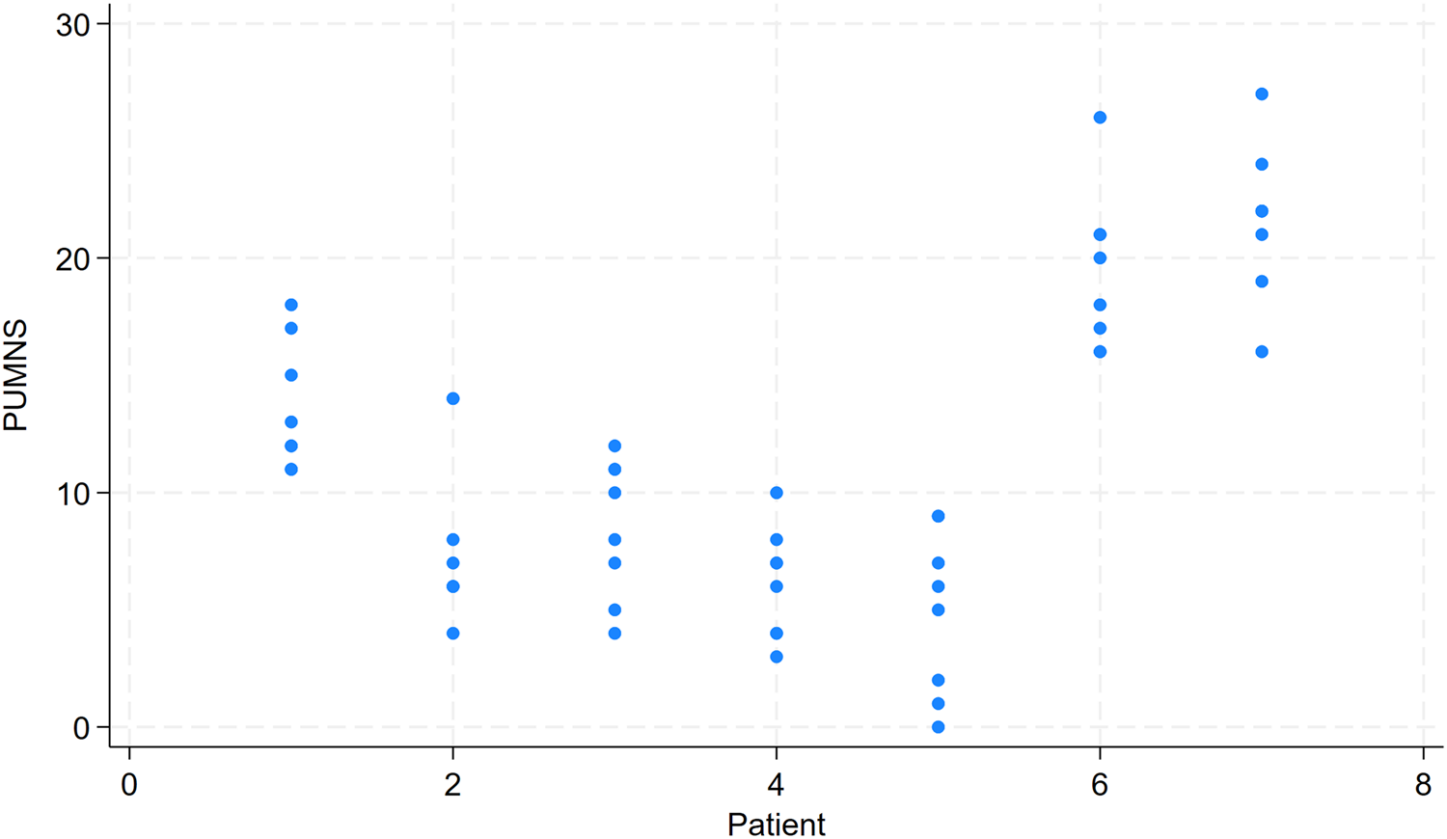
The distribution of Penn Upper Motor Neuron Score (PUMNS) assessed by each rater for each patient

Figure 1 illustrates the total PUMNS given by each of the eight raters, with each line representing a different patient. The inter-rater agreement for the total PUMNS across all raters yielded an ICC of 0.81 (95% CI [0.56;0.96]). Figure 2 and Table 1 illustrate the distribution of PUMNS assessed by each rater for each patient. Notably, the highest standard deviations were observed for patients two and five, indicating significant variability in the evaluations provided by different raters for these patients.

Table 2 presents the inter-rater agreement for each of the subscores, expressed with Gwet’s AC1 coefficient, which provides a general sense of how well raters agree beyond chance. It can obtain a value between −1 and 1. The higher the value, the stronger the agreement between raters. The item with the lowest inter-rater agreement was the facial reflex, with values below 0, indicating that the agreement between raters was worse than what would be expected by chance. Only six out of the eight raters evaluated this item, as it was unclear for the two remaining raters what was meant to be a facial reflex. Other items with low inter-rater agreement included the palmomental sign, crossed adduction reflex, finger flexor reflexes and spasticity. The items with the highest inter-rater agreement were Hoffman's sign, Babinski's sign, and the deep tendon reflexes (biceps, triceps, patella, and ankle reflexes), as well as clonus and jaw jerk reflex. Clonus in the upper limbs received a perfect agreement score of 1, likely because it is a rare finding and none of the raters observed it in any of the patients. The brachioradialis reflex (which is not part of the original PUMNS) was included as an extra subscore and also showed a good agreement between raters.

**Table 2:** Gwet’s AC1 coefficient for each of the subscores in Penn Upper Motor Neuron Score (PUMNS)

	Gwet’s AC1 (CI)	P-value
Jaw-jerk reflex	**0.549** (0.376,0.722)	1.202e-04*
Facial reflex	**−0.038** (**−**0.252,0.175)	6.615e-01
Palmomental sign	**0.312** (**−**0.074,0.698)	4.750e-02*
Triceps reflex right	**0.692** (0.197,1)	7.086e-03*
Triceps reflex left	**0.496** (**−**0.062,1)	3.632e-02*
Biceps reflex right	**0.612** (0.161,1)	7.975e-03*
Biceps reflex left	**0.438** (**−**0.141,1)	5.682e-02
Brachioradialis reflex, right	**0.677** (0.166,1)	8.790e-03*
Brachioradialis reflex, left	**0.471** (**−**0.18,1)	6.351e-02
Finger flexor reflex right	**0.340** (−0.145,0.825)	6.860e-02
Finger flexor reflex left	**0.280** (**−**0.174,0.734)	9.094e-02
Hoffman’s sign right	**0.656** (0.193,1)	6.691e-03*
Hoffman’s sign left	**0.638** (0.232,1)	4.251e-03*
Clonus UL right	**1.0** (1,1)	0*
Clonus UL left	**1.0** (1,1)	0*
Spasticity UL right	**0.444** (0.05,0.838)	1.644e-02*
Spasticity UL left	**0.352** (**−**0.008,0.713)	2.700e-02*
Patellar reflex right	**0.476** (0.106,0.846)	9.956e-03*
Patellar reflex left	**0.668** (0.269,1)	3.207e-03*
Crossed adduction right	**0.177** (**−**0.316,0.67)	2.069e-01
Crossed adduction left	**0.189** (**−**0.311,0.688)	1.954e-01
Ankle reflex right	**0.679** (0.242,1)	4.492e-03*
Ankle reflex left	**0.547** (**−**0.007,1)	2.601e-02*
Babinski’s sign right	**0.547** (0.063,1)	1.633e-02*
Babinski’s sign left	**0.602** (0.188,1)	5.967e-03*
Clonus LL right	**0.915** (0.684,1)	3.428e-05*
Clonus LL left	**0.860** (0.569,1)	1.769e-04*
Spasticity LL right	**0.344** (**−**0.068,0.756)	4.348e-02*
Spasticity LL left	**0.353** (0.125,0.581)	4.562e-03*

*UL* upper limb, *LL* lower limb

### Raters’ experience

After the examinations, each rater completed a questionnaire about how they performed each element of PUMNS, their interpretation, and whether they found it relevant and reliable for UMN assessment in ALS. Only one rater considered the facial reflex relevant, and one found it reliable. Three raters thought the palmomental sign was relevant, while six thought it was reliable. All raters agreed that the triceps, biceps, patella, ankle, the jaw jerk reflex and Hoffman’s reflexes were both relevant and reliable. For the remaining elements, the majority found them both relevant and reliable. The subscore items with the lowest inter-rater reliability were the facial reflex, palmomental sign, crossed adduction, finger flexor reflexes and spasticity. To understand this better, we examined raters' responses to these items in detail. For the facial reflex, only six raters provided answers. Four raters stimulated the perioral area, one stimulated both the perioral area and the forehead, and one stimulated the forehead and cheek. All six agreed on interpreting contraction of the orbicularis oris muscle as a positive reflex, but one rater also interpreted the repeated blinking of the eyes as a positive sign when stimulating the forehead. For the finger flexor reflex, four raters stimulated the fingers, two the palm, and two the wrist. All but one used a reflex hammer to stimulate and they all interpreted flexion of the fingers as a positive reflex. For the palmomental sign, six raters used a wooden stick and two used their fingers to stimulate the palm, with four specifying the thenar region. All agreed that a contraction of the mentalis muscle indicated a positive result. For the crossed adduction reflex, four raters tapped the medial part of patella, one the medial part of the knee, and three the medial part of the thigh at the adductor tendons. Most agreed that adduction of the contralateral thigh indicated a positive reflex. For spasticity, all raters used the modified Ashworth Score, but their interpretations varied significantly. Five raters used a scale from 0 to 2, one rater used 0 to 3 and two raters used 0 to 4, and the definitions for each level differed among raters, ranging from "normal tone" to "mild," "moderate," "severe," and "rigid." When examining the other subscores, specific questions revealed disagreements between raters. Four raters felt a more detailed scale for deep tendon reflexes was necessary, while four disagreed. Three raters interpreted a positive Tromner's reflex as a positive Hoffman's sign, but five did not. Opinions on pathological clonus also varied: one rater cited over 30 s of contraction, two required three beats, one required two to three beats in lower limbs and two in upper limbs, three required more than three beats, and one described it as "inexhaustible."

## Discussion

In this study, we evaluated the inter-rater reliability of the PUMNS which is one of the most commonly used tools to quantify the UMN burden in ALS, among eight raters. When considering the total PUMNS of each of the raters, we found an ICC of 0.81 (95% CI [0.55;0.96]) indicating a good inter-rater reliability. However, when considering the subscores on the scale, we observed considerably higher variability between raters.

As mentioned in the introduction, the reliability of the PUMNS has only been evaluated in one previous study [19]. The study set up was different with two raters and they did not rate each of the subscores but grouped them into bulbar, cervical and lumbosacral segments so the results are not directly comparable. The inter-item reliability for each segment with Cronbach alpha was as follows: bulbar 0.69, right cervical 0.85, left cervical 0.81, right lumbosacral 0.75, and left lumbosacral 0.68. The higher inter-observer reliability reported in this study compared to our study could be explained by the fact that it involved only two raters from the same center. Additionally, one of the raters, who had no prior experience with the scale, received a demonstration before use, which likely also contributed to the higher inter-observer reliability. Another previous study compared PUMNS with the Massachusetts General Hospital (MGH) UMNS scale, which is a scale that only evaluates reflexes not spasticity or pseudobulbar affect, to assess their associations with neuroimaging changes in ALS [23]. The MGH UMNS scores reflexes from 0–4: 0 for absent, 1 for diminished, 2 for normal, 3 for pathological brisk (or retained in MRC 2 or less), and 4 for clonus. Both high and low scores are abnormal. A normal score is 20, but patients with multiple UMN findings can also score 20 if other reflexes are absent. In the study, they found that hyperreflexia was the most prevalent UMN sign and showed the strongest correlation with UMN neuroimaging changes compared to the other subscores of PUMNS. The authors concluded that a UMNS focusing solely on hyperreflexia is clinically relevant and adequate for predicting neuroimaging abnormalities in ALS. Another study evaluated the ability of three UMN scales (PUMNS, UK UMN scale, and MGH UMN scale) in tracking disease progression in PLS patients [14]. The UK UMN scale ranges from 0–15 with points given for abnormal reflexes in the triceps, biceps, brachioradialis, patellar, and Achilles, as well as for Hoffman’s and Babinski signs, and the presence of the jaw jerk reflex. The results indicated that all three scales showed modest increases in scores during the first 7.8 years of symptoms, after which the scores plateaued. For some patients, this plateau occurred near the maximum score, suggesting a ceiling effect. This issue was particularly notable with the UK UMN scale, where many patients achieved the maximum score of 15. The authors concluded that more sensitive measures are necessary for clinical trials to detect the relatively small annual changes in these scores.

### Raters’ experience

The low inter-rater reliability for the facial reflex, palmomental sign, crossed adduction, and finger flexor reflexes were consistent with the feedback provided by the raters, who reported significant variability in how they performed these elements of the PUMNS. For instance, differences in the precise location and method of stimulation likely contributed to the inconsistencies in scoring, even though the raters tended to have similar interpretations of the responses they observed. For spasticity, the raters had very different interpretations of the modified Ashworth Scale, which could explain the low inter-rater reliability. The results are similar to those of a review by Meseguer-Henarejos et al., which assessed the inter- and intra-rater reliability of the Modified Ashworth Scale across 33 studies involving 1,065 patients with spasticity [24]. They reported a comparable level of inter-rater agreement for upper limbs with kappa coefficients, κ + = 0.360 (95% CI 0.241 and 0.468) but a higher agreement for lower limbs κ + = 0.625 (95% CI 0.350 and 0.801).

The triceps, biceps, brachioradialis, patella, ankle reflexes, jaw jerk reflex, Babinski’s and Hoffman’s sign demonstrated high inter-rater reliability. This high level of consistency aligns well with the raters' feedback that these measures were both relevant and reliable for assessing UMN dysfunction in ALS patients. The inter-rater reliability of Babinski's sign has also been assessed in previous studies, with conflicting results. In a study by McCance et al., two observers examined the plantar responses of 41 psychogeriatric patients on two occasions [25]. The observers agreed 6 out of 10 times, with only a 50% chance of eliciting a second extensor response after obtaining one initially, and an 80% chance of eliciting a second flexor response. Another study involving 62 patients from a neurology department, tested by three observers who classified the plantar reflexes as extensor, flexor, or equivocal, found weak inter-observer agreement, with a kappa coefficient of 0.38 [26]. Other studies showed higher inter-rater reliability comparable to this study [27–29].

### Strengths and limitations

The strengths of this study include the participation of eight experienced neurologists from different centers all with many years of expertise in ALS. The study was conducted over a single day, with raters blinded to each other's scores. Additionally, patients were not on antispastic medications that could obscure symptoms. There were also limitations to address. First, the number of patients was limited. Second, the patients did get tired during the examinations, which could potentially affect their cooperation towards the end, consequently making it more challenging for the last raters to evaluate them. However, the order of raters was randomized, and patients were offered breaks between each examination. Third, muscle atrophy was not objectively scored in patients but was instead considered subjectively by each observer when evaluating the reflexes.

### Clinical implications

We found that while the inter-rater reliability of the total PUMNS was good, the reliability for some of the subscores was no better than chance. This discrepancy suggests that even though raters might arrive at similar total scores, the specific subscores they assign can vary widely. One limitation of the UMNS, mentioned in previous studies, is the presence of ceiling effects, which makes it difficult to detect subtle changes in disease progression. This issue is especially pronounced in UMNS with a low range, such as the UK UMN scale (0–15) [14]. Moreover, the progression of UMN involvement in ALS can be slow, leading to significant changes in UMN scores that occur too slow to be captured within the typical time frame of a clinical trial [16]. As Babu suggests [8], it is unlikely that a single UMN scoring system can overcome all limitations and fulfill all requirements. Therefore, it should be considered an additional diagnostic tool to be assessed alongside other clinical data.

Based on our results from this study, we call for an updated UMNS. We suggest omitting or revising the elements with a poor inter-rater reliability, which includes the facial reflex, the palmomental reflex, the finger flexor reflex, the crossed adduction and spasticity. Given the various interpretations of the modified Ashworth Scale, we propose that its reliability could be improved by making it binary, indicating either "present" or "absent." The facial reflex could be replaced by the snout reflex. The proposed changes to the scoring system would enhance consistency with the current diagnostic Gold Coast criteria, which considers one of the following a sign of UMN dysfunction; increased deep tendon reflexes, the presence of pathological reflexes, including Hoffman sign, Babinski sign, crossed adductor reflex, or snout reflex and spasticity [1]. Our suggestions need to be tested in a larger cohort of patients, and hopefully they will enhance the reliability and consistency of ALS research.

## Supplementary Information

Below is the link to the electronic supplementary material.

Supplementary file1 (DOCX 792 KB)

Supplementary file2 (DOCX 34 KB)

## Data Availability

The data that support the findings of this study are available from the corresponding author, upon reasonable request.
