# Supplementary figures and images for: Variability of the Penn upper motor neuron score in amyotrophic lateral sclerosis: need for a revised score
Source: J Neurol. 2025 Feb 15;272(3):208. doi: 10.1007/s00415-025-12895-7 (PMC11829849; doi:10.1007/s00415-025-12895-7)

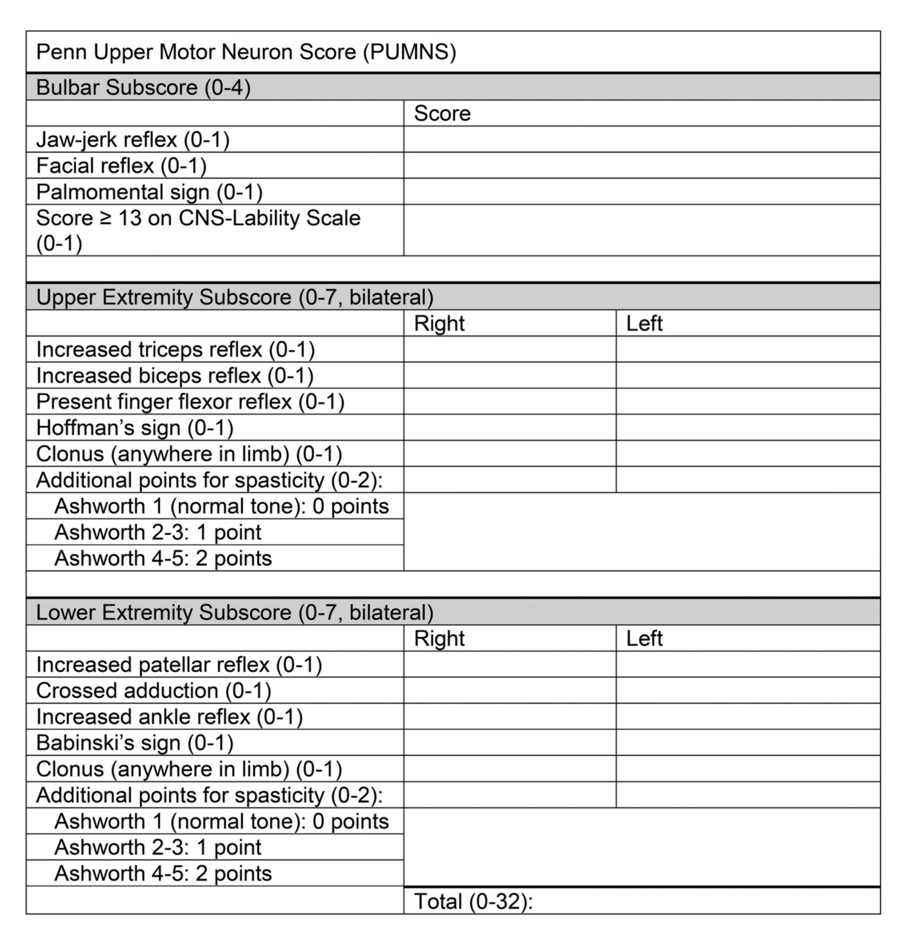

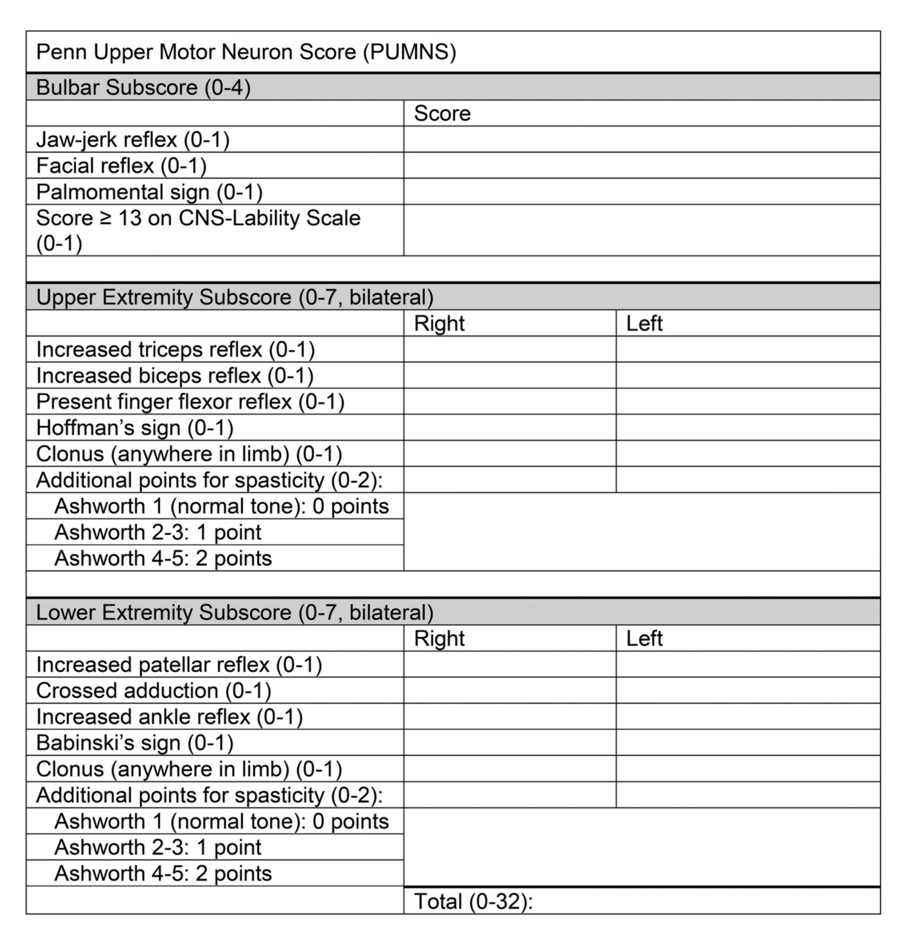

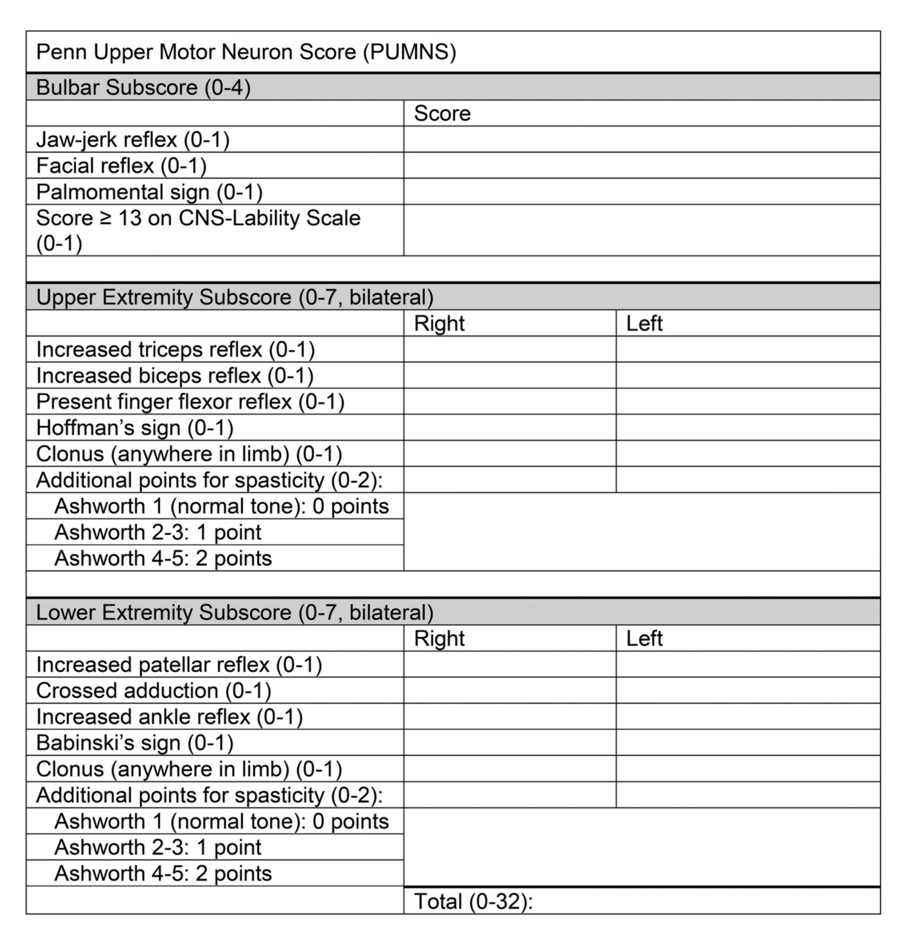

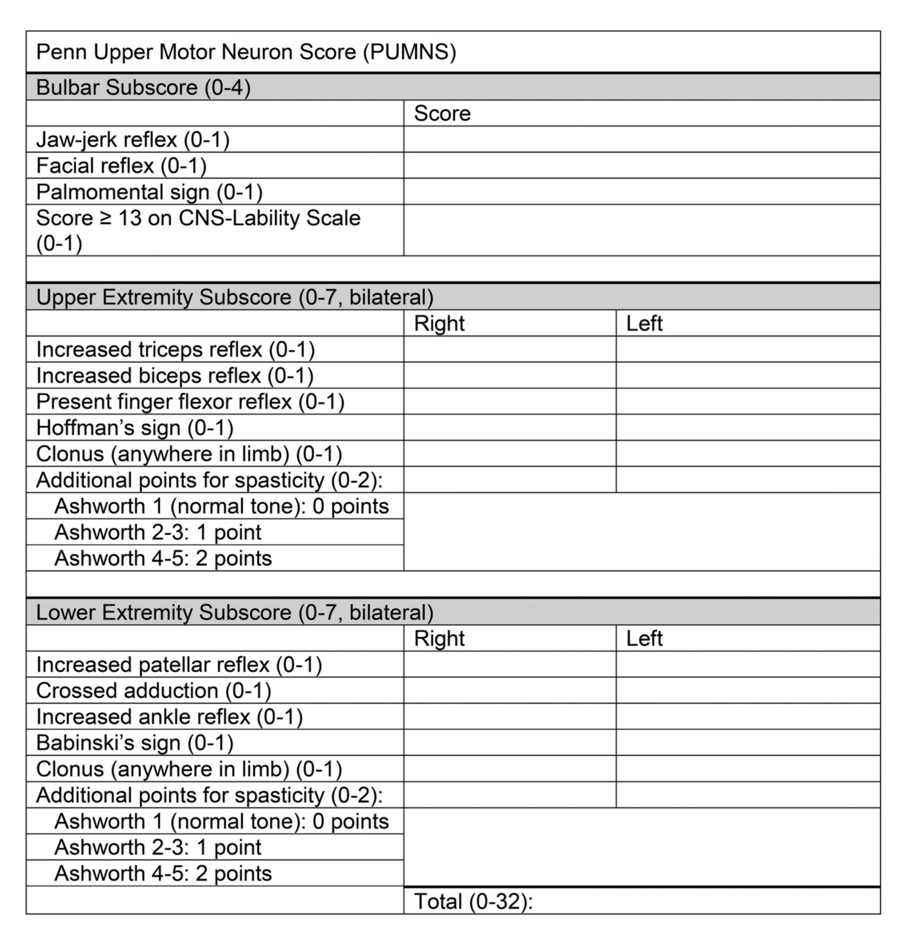

Supplement: Supplementary file 1 — Supplementary file1 (DOCX 792 KB) [file 415_2025_12895_MOESM1_ESM.docx]
